# In Vivo Comparison of Synthetic Macroporous Filamentous and Sponge-like Skin Substitute Matrices Reveals Morphometric Features of the Foreign Body Reaction According to 3D Biomaterial Designs

**DOI:** 10.3390/cells11182834

**Published:** 2022-09-11

**Authors:** Friedrich Barsch, Andreas Mamilos, Volker H. Schmitt, Maximilian Babel, Lina Winter, Willi Wagner, Hinrich Winther, Christian Ottomann, Tanja Niedermair, Stephan Schreml, Helmut Hierlemann, Christoph Brochhausen

**Affiliations:** 1 Institute of Pathology, University Regensburg, 93053 Regensburg, Germany; 2 Medical Center, Faculty of Medicine, Institute for Exercise and Occupational Medicine, University of Freiburg, 79106 Freiburg, Germany; 3 Department of Cardiology, University Medical Centre Mainz of Johannes Gutenberg University of Mainz, 55131 Mainz, Germany; 4 Central Biobank Regensburg, University Regensburg and University Hospital Regensburg, 93053 Regensburg, Germany; 5 Department of Diagnostic and Interventional Radiology, University Hospital Heidelberg, 69120 Heidelberg, Germany; 6 Translational Lung Research Centre Heidelberg (TLRC), German Lung Research Centre (DZL), 69120 Heidelberg, Germany; 7 Institute for Diagnostic and Interventional Radiology, Hannover Medical School, 30625 Hannover, Germany; 8 Centre for Severe Burns with Plastic Surgery, Unfallkrankenhaus Berlin, 12683 Berlin, Germany; 9 Department of Dermatology, University Medical Centre Regensburg, 93053 Regensburg, Germany; 10 PolyMedics Innovations GmbH, 73770 Denkendorf, Germany

**Keywords:** skin substitutes, matrix design, foreign body reaction, histopathology

## Abstract

Synthetic macroporous biomaterials are widely used in the field of skin tissue engineering to mimic membrane functions of the native dermis. Biomaterial designs can be subclassified with respect to their shape in fibrous designs, namely fibers, meshes or fleeces, respectively, and porous designs, such as sponges and foams. However, synthetic matrices often have limitations regarding unfavorable foreign body responses (FBRs). Severe FBRs can result in unfavorable disintegration and rejection of an implant, whereas mild FBRs can lead to an acceptable integration of a biomaterial. In this context, comparative in vivo studies of different three-dimensional (3D) matrix designs are rare. Especially, the differences regarding FBRs between synthetically derived filamentous fleeces and sponge-like constructs are unknown. In the present study, the FBRs on two 3D matrix designs were explored after 25 days of subcutaneous implantation in a porcine model. Cellular reactions were quantified histopathologically to investigate in which way the FBR is influenced by the biomaterial architecture. Our results show that FBR metrics (polymorph-nucleated cells and fibrotic reactions) were significantly affected according to the matrix designs. Our findings contribute to a better understanding of the 3D matrix tissue interactions and can be useful for future developments of synthetically derived skin substitute biomaterials.

## 1. Introduction

Biomaterial matrices are widely used in the fields of skin tissue engineering and regenerative medicine. The central idea of these artificial 3D membrane constructs is to mimic the physicochemical and biological properties of the natural extracellular matrix (ECM) of the skin to create a favorable environment for settling cells and restoring coverage of dermal wounds [1,2]. In this context, biomaterial matrices (=scaffolds) represent three dimensional frameworks that give structural support to cells during the regeneration of full-thickness wounds [3,4]. Based on their sources, the constructs can be classified in natural-derived and synthetic matrices [5,6]. Natural-derived matrices are mostly from allogenic, xenogenic or plant origin, but there are also experiences with marine-derived biomaterials [7,8]. Collagen, elastin, glycosaminoglycans, fibronectin, hyaluronan, gelatin, laminin, chitosan, hydroxyapatite, alginates, silk, pullulan, cellulose and several other polypeptide-based or polysaccharide-based polymers are in use to synthesize skin substitutes [9,10,11]. Due to their occurrence in the native ECM, natural-derived matrices are considered to be better applicable than synthetic biomaterials. Indeed, products from natural-derived biomaterials present a high biocompatibility, due to their low toxicity, low chronic inflammatory responses, good signaling properties and good cell-matrix interactions [9,12,13,14,15]. Unfortunately, natural-derived constructs often failed the quality claims for skin substitutes, due to poor mechanical strength, rapid biodegradation, graft instability and the inability to fix the constructions with sutures on the wound sites. Furthermore, disease transmissions, immunogenicity through antigenic components and high production costs are well known limitations [9,12,13,15]. Therefore, a number of biodegradable synthetic biomaterials were developed. Polyhydroxyorthoesters, such as polyglycolide, polylactide, poly(lactide-co-glycolide) and polycaprolactone are widely in use, due to their favorable properties and excellent biocompatibility. In addition, polyurethane, polypropylene, polyvinylalcohol, trimethylencarbonate and several other synthetics were established [9,11,13,15]. The advantages of synthetic biomaterials are mainly characterized by the favorable controllability of their matrix parameters [16]. For example, mechanical and geometrical properties, such as material strength, stiffness, surface topography, elasticity, porosity, permeability, viscosity, plasticity, stability, crystallinity, molecular weights, as well as, resorbability and degradation rates can be customized [9,14]. The creative modelling of any matrix architecture from a variety of synthetic polymers in combination with reproducible methods guarantees precise manufacturing of large quantities with favorable low costs, low batch variations and low immunogenicity or disease transmission risks [13,15,17,18,19]. Thus, a wide variety of different synthetic matrix designs emerged at the macro, micro, and nano levels, which can be categorized based on their shape [20]. On the one hand, there are fibrous forms like meshes, fibers or fleeces. On the other hand, there are porous forms like sponges and foams [21]. According to the recent literature, the architectural particularities of fibrous or spongy matrices were addressed in several separate studies, but comparative observations of the in vivo tissue reactions between these biomaterial forms are extremely rare. It has been shown that the mechanical characteristics and chemical compositions of these templates significantly influence the cellular reactions [22,23,24,25,26,27]. Subsequently, the cell behavior can be guided by the synthetics, to promote cell migration, attachment, mobility, proliferation, differentiation, spreading, quiescence, self-renewal and even apoptosis [17,26,28,29]. Depending on their design, the constructs can contribute to wound healing by supporting tissue ingrowth and angiogenesis. In this context, the 3D architecture of a biomaterial matrix is able to minimize wound contraction and to promote the transfer of oxygen and nutrients through the constructs [12,23,30,31]. These topics are crucial for the development and the performance of tissue-engineered skin substitutes, as the desired outcome is a proper functional and structural integration of the biomaterial into the host tissue. In this context, synthetic biomaterials have limitations regarding their degradation products, a lack of cell recognition signals, unknown long term effects, prolonged inflammatory responses and potential infections [11,32,33,34]. Furthermore, synthetic biomaterials are associated with foreign body reactions (FBRs) that can negatively influence the overall outcome of a skin substitute [6,15]. The FBR represents a chronic immunologic host response that happens at the tissue biomaterial interphase and persists over the in vivo lifetime of a biomaterial [35,36,37,38,39]. Similar, but not equal to the steps of wound healing, the implantation of a biomaterial into a host induces a multistep cascade of overlapping tissue responses [40]. These processes are complex and can proceed differently [41,42]. On the one hand, there are mild FBRs with an adequate long-term incorporation of the biomaterial as it is seen, for example, with medical sutures [43]. On the other hand, there are strong FBRs with rejections or excessive encapsulations of the implants. The course of these processes is hard to predict, and influencing factors are not yet fully understood, especially with respect to the three-dimensional (3D) matrix design. In this context, the knowledge about the interactions of 3D architectures and the FBR is still incomplete and needs better understanding to optimize the outcomes for synthetic biomaterials [44,45,46]. Furthermore, the assessment of soft tissue reactions in large animal models is rare, and little is known about how 3D features of synthetic, co-polymerized biomaterials influence the host responses in vivo [35,45]. Especially, the differences between synthetically derived filamentous fleeces and macroporous sponges are unknown.

Therefore, the aim of this study was to compare two synthetic, co-polymerized biomaterial morphologies histopathologically, regarding their in vivo FBRs after subcutaneous implantation. For this purpose, analyses of cellular variables of the FBR were performed. We focused on the question to what extent macroporous filamentous fleeces and macroporous sponge-like scaffolds influence responding processes of the FBR. The explored processes include the inflammatory response on the basis of polymorph-nucleated cells (PMNs), the occurrence of foreign body giant cells (FBGCs) and the fibrous reactions. The histomorphological parameters were chosen based on the work of Aamodt et al., who described the inflammatory reaction, FBGC formation and fibrosis to be the most common variables to assess the severity of the FBR [35]. In addition, we put the mutual influences of the evaluated variables in relation with each other in order to deepen the understanding of their interaction during the FBR, dependent on different 3D biomaterial designs. 

## 2. Materials and Methods

### 2.1. Biomaterial Matrices

**Figure 1:**
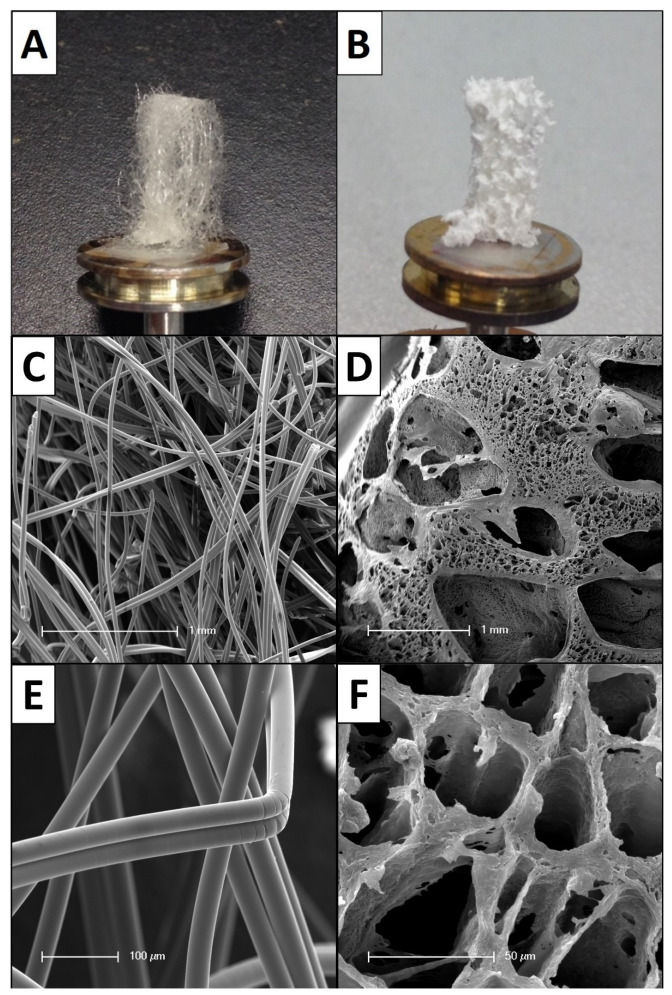
Architectural differences of the biomaterial forms. (**A**) Macroscopic image of a filamentous fleece on a metal carrier. (**B**) Macroscopic image of the sponge-like biomaterial on a metal carrier. (**C**) SEM overview image of the native filamentous fleece: note the random orientation of the fibers; (**D**) SEM overview image of the native sponge-like biomaterial: note the different pore sizes; (**E**) SEM close up image of the filamentous fleece: note the smooth surface structure of the fibers; (**F**) SEM close up image of the sponge-like biomaterial: note the interconnected pores and the rough surface texture.

The synthetic biomaterials were manufactured and provided by Polymedics (Polymedics GmbH, Denkendorf, Germany). Regarding their different geometrical properties, the constructs were grouped in filamentous fleeces (*n* = 16) and sponge-like scaffolds (*n* = 10) (Figure 1). Both groups represent macroporous 3D designs. The filamentous fleeces are composed of filaments with smooth surfaces that form a randomly organized 3D network. The fiber-fleece structures were synthesized using thermoplastic meltblow technology. The fiber diameters are in a micrometer range and ranged from 10 µm to 60 µm. The pore sizes of these scaffolds are indicated as 40–250 µm. Further mechanical properties of the filamentous fleeces are given with a porosity > 85%, extensibility > 50%, resistance > 0.5 N/mm², modulus < 500 N/mm² and shrinkage behavior <20% in phosphate-buffered saline. Chemically, the fibers were fabricated from semi-crystalline co-polymers of poly(lactide-co-glycolides) (PLGA) + trimethylenecarbonat-ε-caprolactone. The lactide to glycolide ratio was 90:10 and the trimethylenecarbonat-ε-caprolactone proportion was 30%.

The sponge-like matrices are foam-membrane composite copolymers that are also produced from PLGA + trimethylenecarbonat-ε-caprolactone. Geometrically, this form is pervaded by randomly interconnected pores with pore sizes of 50–400 µm in their suprastructure and 3–50 µm in their fine structure. The surface texture of these scaffolds is rough and ragged. Further mechanical properties are given with porosity > 90%, extensibility > 50%, resistance > 1 N/mm², modulus < 500 N/mm² and shrinkage behavior < 20% in phosphate-buffered saline. All polymers are biodegradable and widely used in the field of TE, due to their reliable biocompatibility, non-toxicity and non-immunogenicity. Their degradation rates in vitro are described as mechanical degradation within 4 to 8 weeks.

### 2.2. Study Design and Biomaterial Implantation

**Figure 2:**
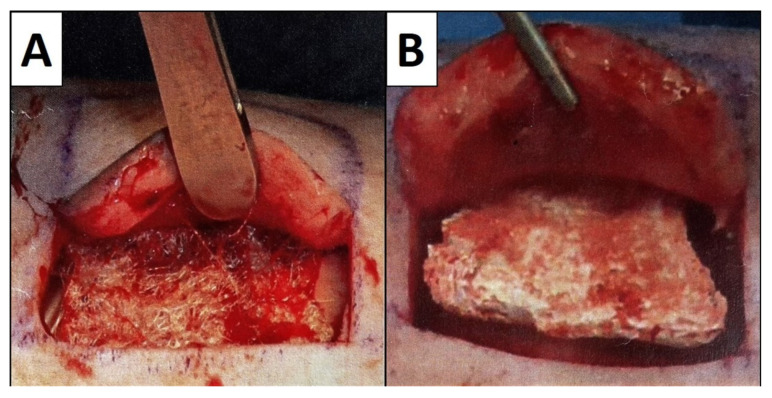
Examples for the subcutaneous implantation procedure of the biomaterial scaffolds. (**A**) Implantation of a filamentous fleece in a skin pocket. (**B**) Implantation of a sponge-like scaffold in a skin pocket.

An animal experiment was performed with 4 European pigs that were used as large animal models to test the tissue engineered products regarding their biomaterial induced in vivo host responses. The setup with pigs has been declared to be an adequate in vivo model, due to the anatomical and histological similarity of porcine skin with the human skin [47]. The animal study was authorized under the reference number V3-2347-A-6-19-2013, in accordance with the European guideline Directive 90/385/EEC and the German provisions of the Animal Welfare Act. Before the animals underwent surgical procedures, subcutaneous implantation regions (test fields) were defined for each animal. The test fields were selected paravertebral on the dorsal sites of the animals and each field measured 4 × 4 cm. The surgical procedures were performed by an experienced surgeon under proper anesthesia of the animals. The animals were premedicated with intramuscular mixed injection of 15 mg/kg ketamine, 0.25 mg/kg midazolam and 0.4 mg/kg azaperone. The animals were intubated and connected to a ventilator. Vital signs were controlled by continuous ECG- and SpO2-monitoring. Proper anesthesia was ensured by continuous infusion of ketamine (10 mg/kg/h) and midazolam (0.5 mg/kg/h) via the ear vein. During operation procedure subcutaneous skin pockets were prepared under sterile conditions. Thereby, the skin was vertically incised at 3 sites of each square skin field and then dissected within the underlying subcutaneous fat layer. Subsequently, a skin flap was created and cautiously unfolded. Then, the biomaterials were randomly implanted to the subcutaneous pockets, with an even distribution between the animals and the implantation regions (Figure 2). To cover the biomaterials, the skin flaps were carefully folded back and occluded with sutures at a sufficient distance from the constructs. Afterwards, the test units were bandaged. The animals frequently got dressing changes during the observation period. On postoperative day 25, the biomaterial scaffolds were completely excised with a narrow hem of surrounding skin tissue. The explanted test fields were immediately immersed in 4% buffered formalin solution. Our pathological institute was commissioned with the histopathological evaluation of the explants.

### 2.3. Sample Processing and Histological Staining

After fixation, remaining bristles were removed with a razor to guarantee an artefact-free histological preparation. From each specimen, two slices of 2–3 mm thickness were vertically taken, one from the center of the explant and one 10 mm in parallel from the first cut. These samples were transferred into tissue cassettes (Kabe Labortechnik GmbH, Nümbrecht-Elsenroth, Germany) and fully automated, embedded in paraffin in the Tissue-Tek^®^ VIP™ processor (SAKURA^®^ Finetek Germany GmbH, Staufen, Germany). Afterwards, 4 µm thick sections were obtained (HYDRAX M55, Carl Zeiss Microimaging GmbH, Jena, Germany), placed on microscope glass slides (Diagonal GmbH & Co. KG, Münster, Germany), dried and deparaffinized. In standardized semiautomated methods, we performed Hematoxylin and Eosin (H&E) staining, as well as Masson-Goldner trichrome staining. H&E-staining was chosen to identify overall architectural tissue particularities. This staining method also allows assessment of tissue ingrowth, neovascularization and inflammatory reactions within the scaffolds. Masson-Goldner trichrome staining is widely used for the analysis of organ fibrosis and for the evaluation of fibrotic responses on biomaterials [35,48,49,50,51,52,53,54,55,56,57,58,59]. This staining also turned out to be beneficial for FBGC assessment. The samples were covered with Entellan (VWR International GmbH, Darmstadt, Germany) in a fully automated glass coverslipper (Leica CV5030, Leica Microsystems GmbH, Wetzlar, Germany). Thus, in total, 104 permanent histological slides were created (52 H&E-stained, 52 Masson-Goldner trichrome stained), which are subject to the same preparation and staining protocols.

### 2.4. Histopathological Analysis

In accordance with modern digital pathology strategies, we based our histopathological analysis on whole slide imaging [60]. For this purpose, all 104 slides were digitized in two automated runs with a digital slide scanner (NanoZoomer 2.0 HT, Hamamatsu Photonics Deutschland GmbH, Ammersee, Germany). The scanning resulted in high quality images (ndpi-format) with a resolution of 456 nm per pixel. The scans were examined regarding their image quality with the scanner-compatible image viewing software NDP.view 2 (Hamamatsu Photonics Deutschland GmbH, Ammersee, Germany). Scanning artefacts and other factors that could influence the performance of our digital image analysis were observed. All scans fulfilled the quality criteria and were declared to be useful for further investigations. All slides showed the structural elements of the biomaterial scaffolds in the subcutaneous zone. This area was set to be our region of interest (ROI). The overall skin architecture of the biomaterial-surrounding tissue was examined. Tissue ingrowth and neovascularization in between the structural elements of the constructs were controlled in a general overview in each slide. The presence of red blood cells within a vascular lumen in between the biomaterial elements was defined as neovascular capillaries. The inflammatory response, the formation of FBGCs and the fibrotic reaction were quantified.

#### 2.4.1. Determination of the Inflammatory Response

**Figure 3:**
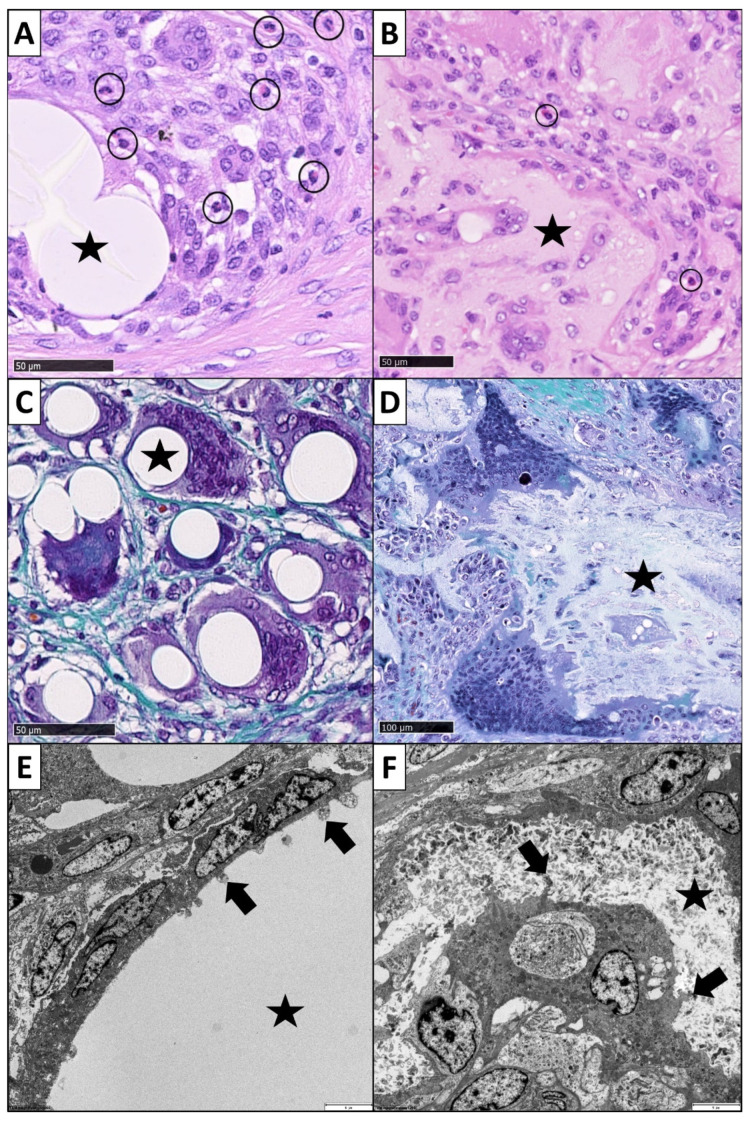
Histopathological analysis of PMNs (black circles) on (**A**) a filamentous biomaterial (star) and (**B**) the sponge-like biomaterial (star). The PMNs clearly show characteristic segmented nuclei (H&E staining, magnification ×400). (**C**) FBGCs in interaction with a filamentous design (star): FBGCs are located in close proximity of the fibers. FBGC shape reminds of an embracement of the fibers (Masson-Goldner trichrome staining, magnification ×400). (**D**) FBGCs in interaction with the sponge-like biomaterial (star): FBGCs can take on gigantic dimensions, show heterogeneous cell shapes and multiple nuclei distributed in the cytoplasm (Masson-Goldner trichrome staining, magnification ×200). (**E**) Ultrastructural TEM image of the interface of FBGC and a filament (star): the cells try to attack the foreign body. Small tongue-shaped cell extensions penetrated the filament surface (arrows), magnification ×1000. (**F**) Ultrastructural TEM image of the interface of FBGC and the sponge-like biomaterial (star): finger-shaped cell extensions can also be seen here, which penetrate into the biomaterial (arrows), magnification ×1200.

To evaluate the inflammatory reaction, we determined the cell density of PMNs as relevant acute inflammation markers (Figure 3) [61,62]. These analyses were performed on the digitized H&E-stained slides with the NDP.view 2 software (Hamamatsu Photonics Deutschland GmbH, Ammersee, Germany). Fifty images per slide at 400× magnification were randomly taken from the ROI. One trained observer (F.B.) manually counted the number of PMNs in each digital image on the basis of typical morphological criteria for this cell type, which were given by a segmented cell nucleus and a spherical cell form with a diameter of about 8 to 15 µm. Their cytoplasm needed to have an eosinophilic to neutral staining color and/or granules. Only PMNs which satisfied these criteria were included into the counting and marked with a circular software tool. The selection of the PMNs and the counting procedure have been performed two times to get adequate results.

#### 2.4.2. Determination of Foreign Body Giant Cells

The measurements of the numbers of FBGCs were performed on digitized Masson-Goldner trichrome stained slides. Similar to the measurements of PMNs, we randomly took 50 images from the ROI at magnification ×400. One trained investigator (F.B.) counted the numbers of FBGCs in each digital image. Cells were identified as FBGCs when they fulfilled the typical morphological criteria. FBGCs were defined as polycaryotic cells with more than 3 nuclei, surrounded by one heterogenic shaped cell membrane (Figure 3). The nuclei had to be uniformly shaped and heterogeneously distributed in the cytoplasm [44,63,64]. We only included FBGCs into the counting if their shape and morphology were clearly identified. The counted cells were marked with a circular tool provided by the software. The cell identification and counting procedure was performed two times to get adequate results.

#### 2.4.3. Analysis of the Fibrotic Reactions

**Figure 4:**
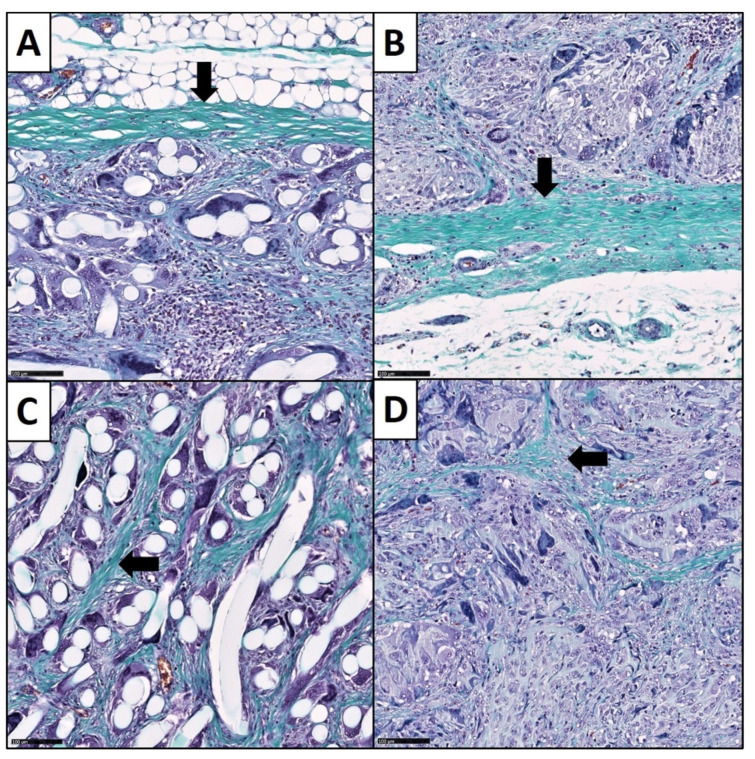
Histopathological evaluation of the fibrotic reactions of the macroporous biomaterials. (**A**) Fibrotic encapsulation (black arrows) around the surface of a filamentous fleece. (**B**) Encapsulation around the surface of a sponge-like biomaterial. Collagen fibers are arranged in parallel and stack in several layers. (**C**) Fibrotic ingrowth within the structure of the filamentous fleece. (**D**) Fibrotic ingrowth within a sponge-like biomaterial. Collagen fibers (arrow) are arranged in diffuse bundles in the space between the architectural elements (Masson-Goldner trichrome staining, magnification ×100).

The fibrotic response on the macroporous scaffolds included the formation of a fibrotic capsule around the whole implant and the fibrotic ingrowth in between the structural elements of the biomaterials (Figure 4). Both reactions were determined with digital image analysis methods, which we established as a valid and quantitative way to analyze the fibrous responses in the context of three-dimensional biomaterials [65]. Therefore, the fibrous capsule thickness (FCT) and the collagen deposition within the matrices (collagen proportionate area = CPA) were measured. We used NDP.view 2 software (Hamamatsu Photonics Deutschland GmbH, Ammersee, Germany) to determine the thickness of the fibrous capsule around the scaffolds in our scans. The software provides a tool which allows measurement of distances in µm. Analogous to Kyriakides et al. we defined 10 measure points around the biomaterial (5 points superficial and 5 opposite points underneath the biomaterial) for each slide [66]. The measurements were performed perpendicular to the long axis through the constructs. At each point we measured the distance of the parallel collagen layers between the biomaterial elements and the surrounding subcutaneous fat. To find the exact boundary layers between biomaterial vs. fibrous capsule and fibrous capsule vs. subcutaneous fat, we performed this process at magnification ×200. To examine the collagen deposition within the matrices, we performed computer assisted, semiautomatic CPA analysis with the open-source image analysis software ImageJ (version 1.49, National Institutes of Health, Bethesda, MD, USA). This strategy is commonly in use for organ fibrosis quantification [67,68,69,70,71]. From each slide, 10 randomly selected images at magnification ×100 were taken. The measurements were performed by a trained investigator (F.B.), under the supervision of experienced pathologists. CPA values are presented as percentages.

### 2.5. Statistical Analysis

All data were calculated using the statistical software IBM SPSS Statistics (version 25.0, IBM Deutschland GmbH, Ehningen, Germany). To examine the distribution of our datasets, we performed One-sample Kolmogorov–Smirnov Tests for each data set. Significance levels were determined with *p* < 0.05. The asymptotic significance (2-sited) was Lilliefors corrected. Subsequently, we performed non-parametric Mann-Whitney U test to compare the two scaffold-groups regarding the evaluated parameters of the FBRs. The significance levels were determined with *p* < 0.05. Furthermore, we determined bivariate correlations between the medians of our variables of each slide. For this purpose, we used Spearman’s rank correlation coefficient with significance levels of *p* < 0.01.

## 3. Results

**Figure 5:**
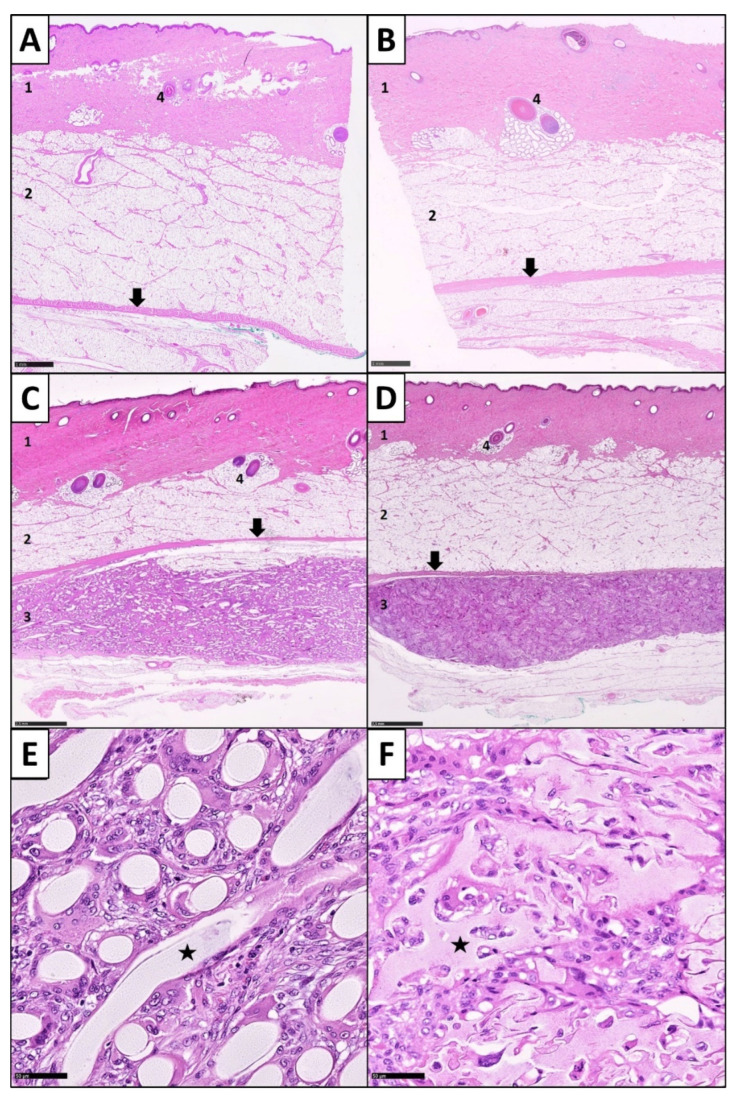
Histopathological evaluation of the overall skin morphometry and biomaterial effects on the surrounding tissue after subcutaneous implantation period (**A**,**B**): Healthy controls of the surrounding skin of the testfields: note the typical zonal morphology: 1 epidermal and dermal layer, 2 subcutaneous fat layer, 4 hair bulges and sweat glands. Arrows point to the fascia superficialis, H&E staining. (**C**) Slide overview after subcutaneous implantation of a filamentous fleece and (**D**) a sponge-like biomaterial. Normal configuration and zonal morphology of the skin with 1 epidermal and dermal layer and 2 subcutaneous fat layers. Below the fascia superficialis (arrow), the biomaterial zone with the implanted scaffolds (3) can be identified, H&E staining. (**E**) Microscopic close-up image of the scaffold zone of a filamentous biomaterial (star), H&E staining, magnification ×200. (**F**) Microscopic close-up image of the scaffold zone of a sponge-like biomaterial (star), H&E staining, magnification ×200.

**Figure 6:**
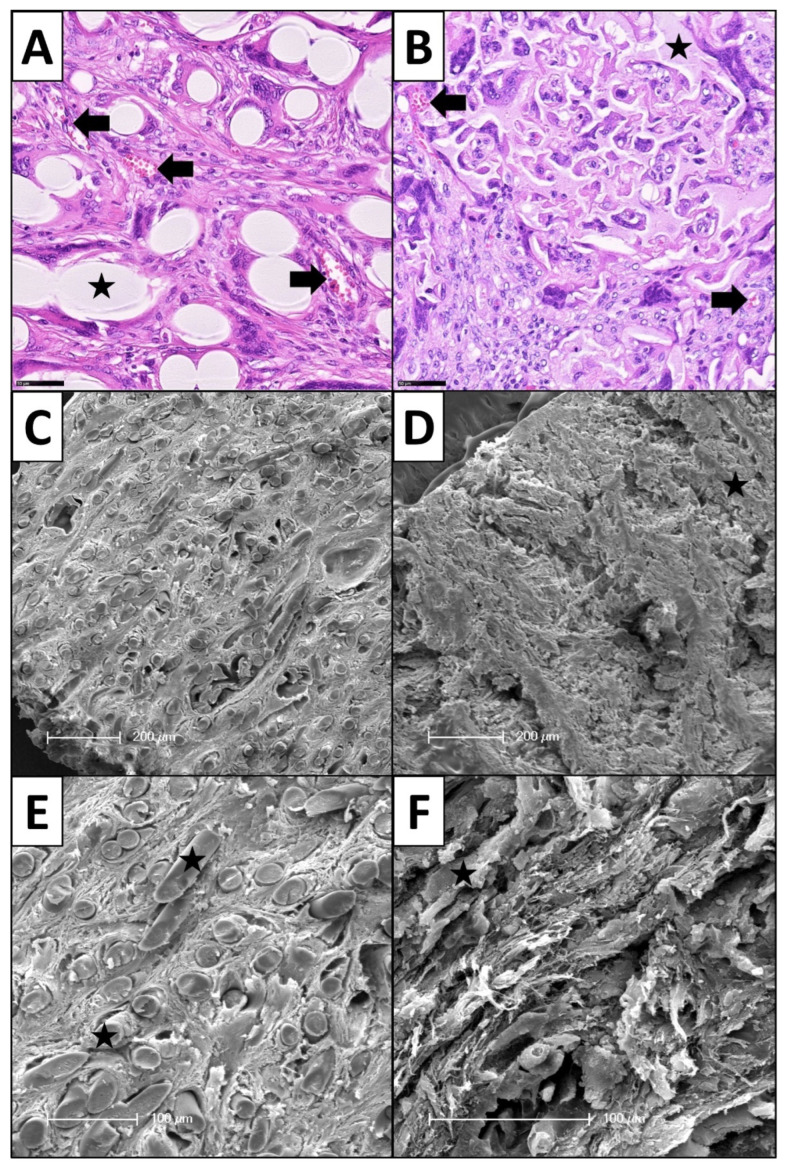
Histopathological and SEM images as examples of tissue ingrowth into the macroporous biomaterial scaffolds during subcutaneous implantation period. (**A**) Neovascularization (arrows) between the fibers of a filamentous fleece (star), H&E staining, magnification ×200. (**B**) Neovascularization in between the spongy elements (star). (**C**,**E**) SEM images of tissue ingrowth in between a filamentous biomaterial (star). (**D**,**F**) SEM images of tissue ingrowth in between a spongy biomaterial (star).

Histologically, after 25 days of subcutaneous implantation, all biomaterials induced an FBR, accompanied with the presence of PMNs, FBGCs, fibrous encapsulation and fibrotic ingrowth. In all samples, the FBR remained limited to the biomaterial in the subcutaneous region. In comparison with healthy controls, superficial dermis, epidermis and subcutaneous fat above the fascia superficialis showed normal stratification and morphological configuration without any tissue damage or inflammatory reactions (Figure 5). All matrices showed a tissue ingrowth and neovascularization in between their structural elements due to their pore sizes (Figure 6). Structurally, the biomaterial elements showed intact morphologies. Only a few pieces showed splits and cracks, but it remained unclear, whether these discoveries were minor signs of degradation or artefacts of slide preparation.

### 3.1. Inflammatory Response

**Figure 7:**
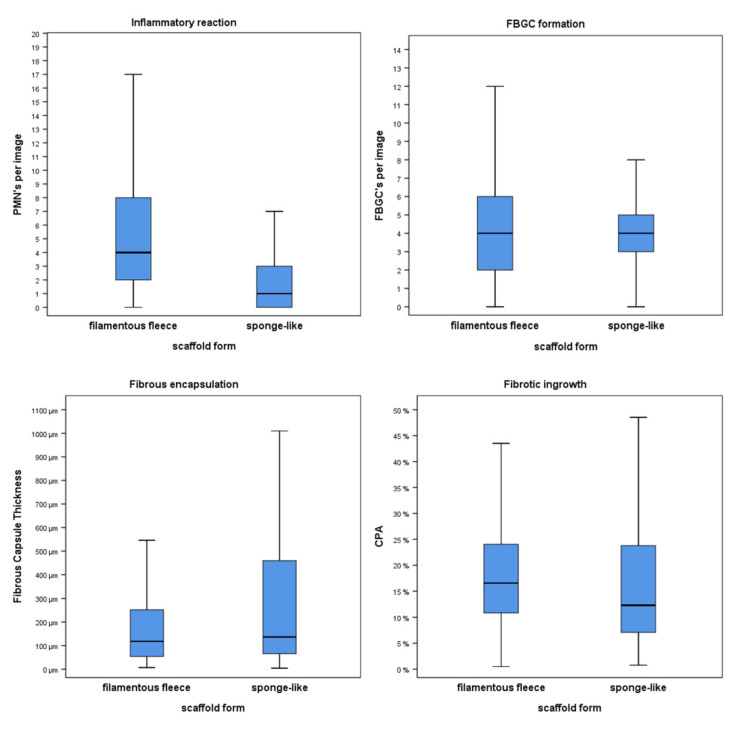
Results of the histopathological comparison between filamentous fleece and sponge-like biomaterial matrices (=scaffolds): variables of the FBR (PMNs, FBGCs, fibrotic encapsulation, fibrotic ingrowth) were quantified and are shown in box plots. Box plots indicate the median and the 5–95% percentiles.

The inflammatory reaction on the 3D biomaterials was quantified based on the PMN cell density. PMNs were localized in between the scaffold elements. The results are shown in Figure 7. The distribution of PMN numbers among the two analyzed biomaterial architectures was significantly different (2-sided test, asymptotic significance, *p* < 0.001). The filamentous fleeces showed 4.00 (2.00–8.00) cells per image, whereas only 1.00 (0–3.00) PMN was found in the sponge-like group. Thus, the number of PMNs per image was higher in the filamentous fleeces compared to the sponge-like synthetics.

### 3.2. Expression of Foreign Body Giant Cells

Histologically, FBGCs were mostly detected in close proximity to the biomaterial surfaces (Figure 3). The cells showed their characteristic heterogenous cell shape with multiple nuclei in the cytoplasm. The filamentous fleeces induced a median number of 4.00 (2.00–6.00) FBGCs per image. The result of the sponge-like constructs was also 4.00 (3.00–5.00) FBGCs per image. Thus, regarding the occurrence of FBGCs, we could not find a significant difference in the number of FBGCs between the two scaffold forms (*p* = 0.178). Nevertheless, the data distribution in the filamentous fleeces had a wider range compared to the sponge-like design.

### 3.3. Fibrotic Reactions

Histologically, the fibrotic reaction showed two different patterns, namely the formation of a fibrous capsule around the entire implants and a relevant fibrotic ingrowth in between the structural elements of the 3D biomaterials. The collagen layers of the fibrous capsule and the collagen fibers of the fibrotic ingrowth could easily be detected due to our staining methods (Figure 4). The fibrotic encapsulation contained parallel-aligned collagen layers on the implant surface. Thus, it was possible to measure the thickness of the fibrous capsule with digital instruments. There was a significant difference between the value distribution of the two matrix forms (2-sided test, asymptotic significance, *p* = 0.016). Here, the filamentous fleeces showed a median capsule thickness of 118.00 µm (53.53 µm–252.00 µm). The FCT measurements of the sponge-like scaffolds showed a median capsule thickness of 136.50 µm (65.40 µm–459.75 µm). Thus, the encapsulation was thicker in the sponge-like group compared to the filamentous fleeces group. In contrast to the aligned collagen arrangement of the capsule, the collagen bundles in between the elements pervaded the interspaces in a diffuse fiber arrangement. Therefore, we quantified the fibrotic ingrowth with the help of digital image analysis (DIA). The evaluation of the CPA was a helpful assessment strategy to compare the different 3D biomaterial forms, regarding their fibrotic ingrowth. The results of these measurements are expressed as percentages of the collagen area in relation to the analyzed image area. The CPA method showed significantly different collagen proportions among the groups (2-sided test, asymptotic significance, *p* = 0.004). The collagen area fraction of 16.55% (10.82–24.06 %) in the filamentous fleeces group was higher compared to the sponge-like group, where we measured a collagen area fraction of 12.32 % (7.05–23.81 %). Consequently, the filamentous fleeces contained a higher fibrotic ingrowth compared to the sponge-like designs.

### 3.4. Correlations between Variables of the Foreign Body Reaction

We further analyzed in what way the numbers of PMNs, the amount of FBGCs, the thickness of the fibrous capsule and the collagen proportion affected each other. For that purpose, we performed analyses of bivariate correlations between all variables with the medians of every slide (Table 1). In the filamentous fleeces group, we found a significant negative correlation between the median numbers of PMNs and the median numbers of FBGCs (*p* = 0.001, r = −0.551). Furthermore, a significant correlation between the median FCT and the median CPA values was found (*p* = 0.006, r = 0.472). Thus, the collagen proportion was higher within the scaffold, in accordance with thicker fibrotic capsules around the scaffold. This could also be demonstrated in the sponge-like group. In this group, we also found a significant positive correlation between the median FCT and the median CPA values (*p* < 0.001, r = 0.738). Beyond that, we could not find any further correlations.

## 4. Discussion

The present histopathological study represents, to our knowledge, the first systematical histopathological comparison of filamentous fleeces and sponge-like biomaterials, with respect to their in vivo influences on the processes of the FBR and fibrosis. The two artificial skin matrix designs were quite similar in their chemical compositions, but differed in their architectural geometry and physical properties, such as the surface topography. Thus, especially the effect of the biomaterial forms on the variables of the FBR could be compared. Based on the numbers of PMNs, the filamentous fleeces induced a significantly higher inflammatory response than the sponge-like architectures. In contrast, FBGC cell numbers showed no significant differences between the two groups. The fibrotic reactions were revealed in the form of an encapsulation around the implants in toto and a fibrotic ingrowth in between the void space of the matrices. In this context, the ratio of the fibrotic reactions differed with a view to the architectures, as the filamentous fleeces showed significantly thinner capsules and significantly more fibrotic ingrowth compared to the sponge-like biomaterials. The histopathologic results of this study are consistent with previous observations, in which the physicochemical properties of biomaterials influenced the FBR [22,23,24,25,26,27]. In this context, it is seen as a challenge to combine the right polymers to create an ideal skin substitute membrane scaffold [24]. The co-polymerization of PLGA with trimethylenecarbonat-ε-caprolactone promotes shape memory behavior and is important for the structural preservation of 3D designs [72]. It maintains further mechanical properties, such as high flexibility, high tensile strength and toughness. In addition, it influences the degradation rate to slower resorption times. The in vivo resorption of large foreign bodies is mainly managed by FBGCs, but synthetic polymers are only slowly engulfed by FBGCs [73]. The degradation of a biomaterial results in the deconstruction of the structural integrity of the biomaterial, however, in turn, the structural integrity is necessary for the mechanical support during the regeneration of soft tissues, which is an existential benefit of scaffold-based skin substitutes. In this context, Lanao et al. summarized that not only the chemical composition, but also the matrix form, strongly affect the degradation rates of biomaterials [74]. Furthermore, Subramanian et al. could show that a large total surface area resulted in higher degradation rates [75]. The two biomaterial forms investigated in the present study provided both a chemical composition and an architectural design that led to favorable long degradation rates. Thus, we were expecting good structural integrity and barely damaged materials after 25 days of subcutaneous implantation. With our histopathological analyses we confirmed this for both groups. Histologically, the biomaterials showed intact structural elements, and thus, a sufficient mechanical stability which would result in the demanded long-term degradation rates. Unfortunately, too long degradation rates are accompanied with stronger FBRs [25]. DiEgidio et al. could show that slowly biodegradable biomaterials showed a prolonged inflammatory phase [76].

### 4.1. PMNs and Their Dependence on the Biomaterial Architecture

From a physiological point of view, acute inflammatory cells are usually only expected during the first week after implantation of a biomaterial. The occurrence of PMNs on day 25 after implantation in both groups indicated a prolonged inflammatory response and thus, by definition, a pathological course of the FBR. Anderson et al. stated that inflammatory phases, that last for longer than 3 weeks within the FBR cascade, can be equated with infections [77]. Interestingly, regarding the numbers of PMNs, the prolonged inflammatory reaction was stronger in the filamentous fleeces group compared to the sponge-like group. This finding is in line with the study of Bygd et al., who found that PLGA substrates with flat surfaces induced a higher pro-inflammatory cytokine profile [78]. In addition, Julier et al. found that surface topography is related to the immunogenicity of a biomaterial [79]. Regarding the inflammatory reaction and the FBR, we suggest, based on our findings, that cell differences occurred due to a different level of the overall severity of the FBR. Furthermore, this observation could be an expression of a different time course of the FBR. In this context, potentially, the filamentous fleeces are still in a more acute inflammatory phase with higher amounts of PMNs, whereas the sponge-like matrices are already merged into a more prolonged state of the FBR, which is pathophysiologically accompanied by the continuous disappearance of PMNs. However, the reasons for the different cell counts remains unclear, since this question could not be addressed by the study design. Furthermore, the assessment of the overall inflammatory response using PMNs as a sole parameter cannot be determined definitively. Here, future studies should aim for a more differentiated observation of inflammatory cells and mediators such as macrophages, lymphocytes, interleukins and tumor necrosis factor alpha (TNFα) to obtain a holistic understanding of the inflammatory processes in the context of different biomaterial designs. Since the purpose of this study was to assess the local tissue responses, no other systemic inflammatory parameters were observed. This can be seen as a limitation of the study, based on the fact that there is a lack of established antibodies for immunohistochemical staining of macrophages or T lymphocytes in porcine tissue [80]. Furthermore, future study designs should address different time points and durations of the single steps of the FBR, including an extensive differentiation of the cellular and humoral markers of the respective inflammatory reactions during the long-term development of the FBR.

### 4.2. Foreign Body Giant Cells in the Context of Synthetic Matrix Designs

From a pathophysiological point of view, there might be a connection between PMNs and FBGCs, as PMNs attract macrophages to the scene via IL-8, MCP-1 and MIP-1ß and thus promote the formation of FBGCs [64]. Physiologically, FBGCs can be seen from the tenth postimplant day for the whole in vivo lifetime of larger biomaterials [63]. Therefore, it was no surprise that there were FBGCs on day 25 after implantation in both groups. The formation of FBGCs is also related to the physical properties of biomaterials. Klopfleisch et al. concluded, according to their findings, that the surface topography influenced the formation of FBGCs, as they found that flat surfaces induced more FBGCs compared to rough surfaces [64]. In contrast to these findings, Anderson et al. described that biomaterials with a rougher surface induced increased layers of macrophages and FBGCs [81]. In our present histopathological evaluation, we could not find significant differences in the number of FBGCs regarding the different surface topographies of the used designs. Furthermore, the discovered negative correlation between PMNs and FBGCs in the filamentous fleeces was unexpected, since we assumed, in accordance with the physiological processes of the FBR, that higher numbers of PMNs would attract more inflammatory cells, such as macrophages and lymphocytes to the scene, and subsequently should result in the formation of more FBGCs. Taking all these findings together, it could not be clarified to what extent the PMN cell numbers, the 3D geometry or the surface topography of the biomaterials influence the FBGC numbers. However, it can be assumed that the FBGC formation is a multifactorial process, whose respective concrete influences are not yet completely understood. In this context, further factors could be of relevance, which unfortunately could not be addressed by our histopathological examination. Here, a further examination of the biochemical interdependencies between PMNs and FBGCs and the specific role of degradation products of the scaffold material would be of special interest. One interesting aspect to analyze in future investigations is the relationship between FBGC shapes and functions in interaction with different biomaterial designs regarding a structure-function paradigm.

### 4.3. Fibrotic Growth Patterns and Their Dependence on Physical Matrix Parameters

Serin et al. concluded that the fibrotic ingrowth represents a successful graft-host response [82]. We agree with this assumption, on the condition that the fibrotic ingrowth does not lead to negative effects on the tissue, but leads to an adequate integration of the biomaterial into the host tissue and supports wound healing processes and a fast coverage of skin defects. Regarding the fibrotic reaction, the present study demonstrated that the macroporous structure of both biomaterial forms induced not only a fibrous capsule around the implants, but also a fibrotic ingrowth in between its structural elements, and that these reactions correlate significantly with each other. Thus, following the assumption of Serin et al., the fibrotic ingrowth would mean accepting thicker encapsulations around the biomaterials. However, in the field of skin tissue engineering, an intense encapsulation is an undesired tissue response. Consequently, the question for macroporous 3D membrane customizing should be how to shift the fibrotic ingrowth vs. fibrotic encapsulation ratio to the fibrotic ingrowth side. In this context, we could demonstrate that the 3D architecture of a biomaterial may be an important influencing factor, as we could detect significant differences in the patterns of the fibrotic responses between the investigated constructs. The fibrous encapsulation was thinner, and the collagenous ingrowth was higher in the filamentous fleeces group compared to the sponge-like group. This difference could be explained with pathophysiological aspects of the FBR and the morphological features of the matrices. The different surface topography seems to modify the fibrotic growth patterns. After subcutaneous implantation, fibrotic effector cells need to migrate from the surrounding tissue in between the structural elements of the biomaterials. In accordance with the theory of Hogrebe et al., who argue that cells have a migration capability along the direction of fibers, which the authors called ‘contact guidance’, we suspect that the rougher surface topography of the sponge-like designs delayed the fibrotic effector cells’ immigration compared to the fibrous matrices. This hypothesis should be tested in future in vitro assays. Cells can adopt their shapes and cytoskeletal organization to the forces and fibers of a fibrous network. Thus, fibrous biomaterials could facilitate the migration of the cells [20]. Christo et al. described that rougher surfaces altered the cell adhesions and cell motilities. Here, the orientation, shape and distortion of surfaces influenced the protein binding and, subsequently, the cell behaviors [83]. In addition, Anderson et al. demonstrated that flat surfaces induced decreased numbers of macrophages and lower fibrotic reactions [81]. Furthermore, Salthouse et al. could show that rougher surfaces profoundly affected the behavior of macrophages, which are in turn influencing the fibrotic effector cells [84]. According the ‘contact guidance’ theory, it can be assumed that cell migration of fibrotic effector cells into a microporous biomaterial is facilitated along smooth filamentous surfaces, compared to rough spongy surfaces. The results of the comparison in the present study confirm this hypothesis. Higher collagen proportions inside, and thinner fibrotic capsules around the filamentous fleeces could be found compared to the sponge-like matrices. Thus, the fibrotic ingrowth vs. encapsulation relation behaves in favor of fibrotic ingrowth in the filamentous scaffolds, which, considering their use as a skin substitute, can be understood as a more advantageous tissue integration.

Beside the surface topography, the pore size of microporous constructs is known as an important parameter that could also lead to different fibrotic growth patterns. The pores of both biomaterial forms used in our study were large enough to enable a sufficient tissue ingrowth, which was also obvious, due to the adequate neovascularization. In this context, it is known that the 3D porous geometry and microenvironment determines the cell migration and proliferation [85]. Feng et al. analyzed decreased fibrotic ingrowth rates with increasing pore sizes of bioceramics [86]. In contrast, Sussman et al. detected decreased fibrosis in hydrogel scaffolds, with 34 µm pore sizes compared to hydrogels with 160 µm pore sizes [87]. Ward et al. described an extreme case: they compared solid materials with porous sponges in a subcutaneous rat model. They found thicker fibrotic capsules and higher capsule densities in the solid material group [88]. Solid biomaterials prevent a cellular immigration into their center, whereas porous materials enable this cell behavior. As a result, the production of ECM proteins of biomaterials with smaller pore sizes and a higher textured fine structure is more likely to take place at the implant-tissue interface than in the center of an implant. So, the differences in the fibrotic growth patterns between the investigated biomaterial forms could also be an expression of the different pore sizes in between the groups. Here, the lower pore size of the fine structure of the sponge-like scaffolds has been shown to play a considerable role in the formation of the fibrotic growth pattern differences. However, pore sizes in the synthetics varied randomly, up to a size of 400 µm. In this context, it could be shown that random pore sizes promote chronic inflammation and enhance the FBR [36]. Our study was able to confirm these findings for the investigated duration of inflammatory responses in both groups.

Another hypothesis regarding the different fibrotic responses is that the different architectures of the biomaterials induced different mechanical stress on the surrounding tissue, since mechanical shear stress between the biomaterial and the surrounding tissue leads to a compensatory remodeling of cell-cell contacts and the ECM [89]. Especially, a mismatch between the material stiffness and the tissue softness induces higher mechanical stress levels and subsequently increases the formation of fibrotic capsules [64]. Higher stress levels would induce thicker fibrotic capsules around an implant, which should be considered for the membrane design in skin tissue engineering. Regarding the material stiffness, a higher number of crosslinks gives the spongy architecture a higher material stiffness compared to the filamentous fleeces. In addition, higher extensibility and a rougher surface with sharper edges given by sponge-like designs contributed to an overall stronger mechanical stress on the surrounding tissue. In contrast, fiber based biomaterials provide filaments with smoother surfaces and a high surface to volume ratio and thus, would distribute the shear stresses more equally through the implants and the tissue which results in reduced cellular signaling [10,89,90]. Based on these theories, we suggest that the sponge-like architecture induced higher mechanical stress on the surrounding tissue and subsequently generated thicker fibrous capsules compared to the filamentous fleeces.

### 4.4. The Importance of Biomaterial Design for the Use as Skin Substitutes

The question arises whether spongy or filamentous designs would be more beneficial for use as synthetic membranes for skin regeneration of deep dermal destructions. In this context, both matrix architectures seem to have their advantages and disadvantages in terms of FBRs. Considering recent study results in the literature, the significance of the biomaterial designs for the use as skin substitutes can be derived. Regarding the filamentous architecture, for example, Venugopal et al. determined that cells grow along the fiber directions and form networks according to the 3D architecture of scaffolds [91]. Chaudhari et al. hypothesized that fiber-based biomaterials would promote cell adhesion and differentiation in a better way than sponges, caused by their higher structural similarity to the natural ECM [21]. Regarding the FBR, Bryan et al. analyzed subcutaneously implanted surgical meshes in a rat model and demonstrated that polyfilaments induced higher amounts of FBGCs compared to monofilaments [92]. Also, Subramanian et al. tested randomly organized PLGA fibers in a subcutaneous rat model and found increased numbers of PMNs and FBGCs [75]. These observations are further supported by the work of Bygd et al. [78]. Furthermore, Cao et al. tested polycaprolactone nanofibers with random orientation in a subcutaneous rat model. They showed that randomly oriented fibers were accompanied by increased healing times, increased monocyte adhesions, higher numbers of FBGCs and chronic inflammation [93]. This is in accordance with our histopathological observations, wherein we found increased numbers of PMNs and an overall prolonged inflammatory response in the randomly organized filamentous group. Thus, regarding the inflammatory reaction, we can summarize that a random fiber orientation is probably unfavorable for use as a skin substitute. On the other hand, Kempf et al. showed that a random fiber orientation prevents implant contraction, which is in turn of special interest in the field of skin tissue engineering [94]. Regarding the fibrotic reactions, Cao et al. demonstrated that randomly oriented fibers showed thinner fibrous capsules and more granulation of tissue compared to aligned fibers [93]. Further, Ogle et al. described that randomly oriented nanofibers resulted in a reduced fibrous encapsulation [45]. In our study, the fibrotic ingrowth and fibrotic encapsulation ratio in the filamentous group were in a favorable relationship, corresponding to a more beneficial tissue integration of skin substitutes.

In contrast, the sponge-like designs induced favorable lower numbers of PMNs, but showed a fibrotic growth pattern that shifted toward fibrotic encapsulation, which can be considered as disintegration in the context of use as skin substitutes. Nevertheless, the spongy architecture can offer a couple of advantages. In this context, Chattopadhyay et al. defined sponges as having a good adherence on the wound bed and support of the tissue regeneration, by maintaining a favorable environment and protection from mechanical trauma or bacterial infection [95]. Furthermore, Chaudhari et al. postulate that sponges give more stability compared to mesh structures [21]. These aspects are supported by the work of Romanova et al., who could show that sponges are resistant to fibroblast contractions and provide a better diffusion [96].

Regarding their use as 3D skin substitute membranes, our results cannot give a final decision about the question of which design would be more favorable. Based on our study design, it is not possible to draw a conclusion as to how the processes of the FBR would have developed over a further time course. In this context, it is known that modifications of synthetic biomaterials could improve, but only little affect the long term FBR outcomes [35]. Ghanaati et al. explored the FBR on subcutaneously implanted bone substitutes. They could show that different FBRs in the beginning converged after 15 days [97]. Nevertheless, we found strong FBR courses in both groups, which is an unfavorable aspect for skin substitute-assisted wound healing. We suggest that this phenomenon can be traced back to the overall synthetic character and long degradation rates of the polymer compositions. Here, further developments of synthetic biomaterials should explore further adaptations of the degradation rates to the tissue ingrowth and tissue remodeling to improve the effectiveness of end products [98]. In addition, several other strategies have been attempted to minimize or even overcome the FBR. Surface modulations, cross linking, hybridization of natural and synthetic biomaterials, inclusion of RGD sequences, stem cell deliveries or coatings with anti-inflammatory drugs are some examples that have been studied with more or less success [45,99]. Thus, there are a lot of adjusting strategies and multiple factors influencing the FBR. In this context, it remains an ongoing challenge to develop suitable synthetic 3D designs for use as skin substitutes.

## 5. Conclusions

In conclusion, this histopathological study could verify that the 3D architecture and physical parameters of synthetic skin substitute biomaterials influence the tissue reactions and courses of the FBR. The filamentous fleeces showed higher inflammatory cell numbers, but a more favorable ratio of the distribution of fibrotic growth patterns compared to the sponge-like designs. Regarding the numbers of FBGCs, the study could not show any difference between the matrix designs, but revealed differences in the cell shape of FBGCs. In this context, further structure-function analyses of FBGCs and biomaterial forms would be worthwhile. Regarding their use as skin substitutes, both material designs would have their advantages and disadvantages, which should be further investigated in context. Thus, it remains an ongoing challenge for the future development of synthetic skin substitute membranes to find new parameters and biomaterial compositions to guide the course of the FBRs in a desired way.

## Figures and Tables

**Table 1:** Bivariate correlations (Spearman) between variables of the FBR regarding the biomaterial groups.

Biomaterial Group	Correlations	Spearman’s r	Significance (2-Tailed)
Filamentous fleeces	PMN & FBGC PMN & FCT PMN & CPA FBGC & FCT FBGC & CPA FCT & CPA	(−)0.551 0.221 (−)0.290 (−)0.250 0.044 0.472	0.001 * 0.224 0.873 0.893 0.810 0.006 *
Sponge-like form	PMN & FBGC PMN & FCT PMN & CPA FBGC & FCT FBGC & CPA FCT & CPA	0.380 0.021 0.065 0.208 0.161 0.738	0.110 0.932 0.792 0.408 0.523 0.000 *

* *p* < 0.01.

## Data Availability

Not applicable.
